# The Rat Grimace Scale: A partially automated method for quantifying pain in the laboratory rat via facial expressions

**DOI:** 10.1186/1744-8069-7-55

**Published:** 2011-07-29

**Authors:** Susana G Sotocinal, Robert E Sorge, Austin Zaloum, Alexander H Tuttle, Loren J Martin, Jeffrey S Wieskopf, Josiane CS Mapplebeck, Peng Wei, Shu Zhan, Shuren Zhang, Jason J McDougall, Oliver D King, Jeffrey S Mogil

**Affiliations:** 1 Dept. of Psychology and Alan Edwards Centre for Research on Pain, McGill University, Montreal, QC H3A 1B1, Canada; 2 Boston Biomedical Research Institute, Watertown, MA 02472 USA; 3 Dept. of Physiology and Pharmacology, University of Calgary, Calgary, AB T2N 4N1, Canada

## Abstract

We recently demonstrated the utility of quantifying spontaneous pain in mice via the blinded coding of facial expressions. As the majority of preclinical pain research is in fact performed in the laboratory rat, we attempted to modify the scale for use in this species. We present herein the Rat Grimace Scale, and show its reliability, accuracy, and ability to quantify the time course of spontaneous pain in the intraplantar complete Freund's adjuvant, intraarticular kaolin-carrageenan, and laparotomy (post-operative pain) assays. The scale's ability to demonstrate the dose-dependent analgesic efficacy of morphine is also shown. In addition, we have developed software, Rodent Face Finder^®^, which successfully automates the most labor-intensive step in the process. Given the known mechanistic dissociations between spontaneous and evoked pain, and the primacy of the former as a clinical problem, we believe that widespread adoption of spontaneous pain measures such as the Rat Grimace Scale might lead to more successful translation of basic science findings into clinical application.

## Introduction

Despite great advances in basic understanding of molecular pain mechanisms and considerable investment by industry, translational achievements in analgesic drug development have been extremely limited. Many believe that the high attrition is due, at least in part, to the poor predictivity of current animal models of pain [1]. As *in vivo *animal research remains the mainstay of analgesic drug development [2,3], much recent effort has been devoted to reexamining pain testing paradigms in laboratory animals. Of the criticisms directed at the *status quo *in rodent algesiometry, one of the most common is that the vast majority of preclinical studies measure withdrawal responses to evoking thermal and mechanical stimuli instead of the more clinically important spontaneous pain [4]. Although a number of rodent behaviors are correlated in time with injuries that presumably also produce spontaneous pain, in many cases it has been difficult to demonstrate that these behaviors display specificity and sensitivity as measures of pain [5].

Because of the known utility of facial coding scales (based on the facial action coding system; FACS) [6] for the quantification of pain in non-verbal human populations [see [7]], and the prediction by Darwin that nonhuman animals exhibit similar facial expressions to emotional states as do humans [8], we recently developed and characterized the Mouse Grimace Scale (MGS) [9]. It consists of five facial "action units" (orbital tightening, nose bulge, cheek bulge, ear position, and whisker change) scored on a 0-2 scale for their prominence in still photographs taken from digital video of mice in either a baseline or pain condition. We demonstrated that the MGS displays high accuracy and reliability, is useful for quantifying pain of moderate duration (from several minutes to approximately 1 day), is sensitive to detecting weak analgesic effects, and may represent a measure of the animal's affective response to pain [9].

The purpose of the present work was two-fold. First, despite increasing use of the mouse over the past few decades, the rat remains by far the most common subject of preclinical pain research [1]. The evolutionary stability of facial expression [7,8] would clearly predict that the MGS could be translated to the rat. Second, the main practical disadvantage of the MGS is the labor-intensive nature of one step in the process: grabbing individual face-containing frames from digital video, which is hampered by uncooperative subjects (not looking directly at the camera) or otherwise poor optics due to motion blurring. The utility of this method would thus be greatly improved by automated frame grabbing. We report here the development of the Rat Grimace Scale (RGS), its ability to quantify pain in three common algesiometric assays (intraplantar complete Freund's adjuvant, intraarticular kaolin/carrageenan, and laparotomy), and the development of Rodent Face Finder^® ^software for automated generation of scoring-ready still photographs of both mouse and rat faces.

## Materials and Methods

In all experiments, male and female rats were used in equal numbers [10]. No sex differences were observed and so data were combined for reported analyses.

### Animals

All subjects were Wistar rats, aged 6-8 weeks (200-250 g), obtained from Charles River Laboratories (Boucherville, QC). Rats were housed in groups of 2-4, under a 12:12-hour light cycle (lights on at 07:00 h) in a temperature-controlled environment (20 ± 2°C) with *ad lib *access to food (Prolab RHM 2500) and tap water. Each nociceptive assay utilized a separate cohort of rats, such that no subject participated in more than one assay. All studies were approved by a local animal care and use committee, and were consistent with national guidelines.

### Inflammatory assays

Inflammatory assays were used in this study since the limited duration of facial grimacing is not appropriate for neuropathic assays. Complete Freund's adjuvant (CFA), kaolin and carrageenan were all obtained from Sigma (St. Louis, MO). In the intraplantar CFA model [11], rats were injected with 50% CFA, in a 150 μl injection volume, into the plantar surface of one hind paw. Rats (*n *= 10) were tested before, and 1 h, 4 h, 6 h, 24 h and (in a separate cohort; *n *= 8) 48 h and 7 days post-injection. In the rat intraarticular kaolin/carrageenan model [12], 2% kaolin and 2% carrageenan were successively injected (separated by 10 min), under isoflurane/oxygen anesthesia, into one knee joint, each in a volume of 200 μl. Rats (*n *= 6) were tested before, and 3 h, 6 h, and 12 h post-injection. Group sizes were based on our experience using similar assays in mice [9].

### Laparotomy

A laparotomy, designed to mimic a sham ventral ovariectomy [13], was performed under isoflurane/oxygen anesthesia. Following shaving and disinfection, a 1-cm midline incision was made using a scalpel. Muscle layers were closed with polydioxanone suture 5-0 (Vicryl^®^; Ethicon, Somerville, NJ) and skin edges apposed using tissue glue (Vetbond^®^; 3M, St. Paul, MN). Rats (*n *= 6) were tested before, and 1 h, 4 h, 6 h and 12 h post-surgery.

### Morphine

Morphine sulfate was obtained from Sandoz Canada. Mice were injected with physiological saline (10 ml/kg) or 1, 2, or 5 mg/kg morphine (*n *= 4-8/dose), administered 5.5 h after CFA (see above) and 15 min before the start of 30-min digital video recording (see below).

### Digital video

Rats (two at a time) were placed on a table top in cubicles (21 × 10.5 × 9 cm high) with walls of transparent Plexiglas^® ^and a separating wall of removable stainless steel. One digital video camera was placed on either side of the apparatus in order to maximize the opportunity for clear head shots. Rats were digitally videotaped using high-resolution (1920 × 1080) digital video cameras (Sony High Definition Handycam^® ^Camcorder; model HDR-CX100) for 30 min immediately prior to injection or surgery (baseline or *no pain *photos), and for 30 min at various time points after injection or surgery (*pain *photos).

### Automated frame capture using Rodent Face Finder^®^

Previous to the development of Rodent Face Finder^® ^(RFF), we extracted images manually from digital video. Using Windows Media Player, individual frames of the resultant AVCHD video files were "grabbed" and cropped (so that body position was no longer visible) using the Windows 7 Snipping Tool whenever a clear, unobstructed head shot was observed. This process is considerably labor-intensive, and a C++ program (using the Open CV2.0 library; http://opencv.willowgarage.com), RFF was developed to automate it.

**Figure 1:**
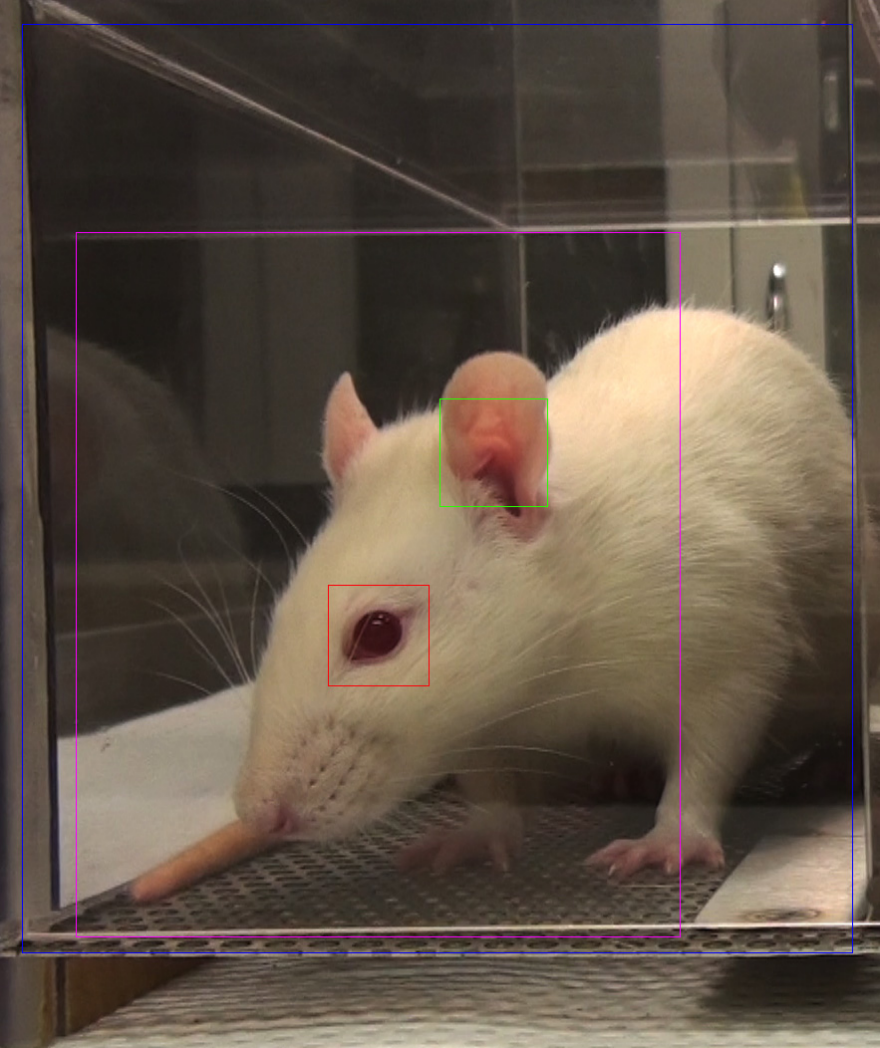
**Uncropped image identified by RFF for RGS scoring**. Boxes: blue, total region analyzed; red, detected eye; green, detected ear; purple, estimated face region.

RFF detects rodent eyes and ears using boosted cascades of Haar classifiers [14], which use differences between pixel intensities in small rectangular regions (Haar-like features) to capture textural and orientation information, and combine the response from many such regions to make predictions on whether a specific sub-region in an image contains an eye or ear. The precise regions and cutoffs used by the cascades were obtained by Haar training, using approximately 500 cropped images of ears and eyes from both baseline and pain-experiencing rodents as positive examples, and a comparable number of non-face-containing frames and unrelated images as negative training examples. The resulting detectors were used to scan each video frame for eyes and ears, at a variety of scales. Frames with at least one eye and at least one ear detected, and satisfying bounds on the distance between them to reduce false positives, were flagged as candidates for scoring. Figure 1 illustrates a video frame flagged by RFF for scoring.

To reduce the number of images for manual scoring, and to minimize blurring due to rapid motions such as grooming, among all the candidate video frames in each 3-min time interval, only the three with the smallest average absolute pixel difference relative to the previous video frame (1/30^th ^of a second earlier) were saved as images. Both the feature detection and motion estimation were restricted to zones of the video corresponding to single cages, with the left and right cages analyzed separately.

From each 3-min time interval, the single image most suitable for manual RGS scoring was manually selected from among the candidate images. For some intervals, no candidate images were extracted by RFF, which can occur if the rodent does not face either camera during this interval, or due to false negatives in the program. In some intervals with no candidate images, images were extracted manually.

### RGS coding

Image files were then copied into PowerPoint, one image per slide. A PowerPoint macro (http://www.tushar-mehta.com/powerpoint/randomslideshow/index.htm) was then used to randomize the slide order. Identifications were removed in order to ensure that subsequent coding was performed blind.

Randomized and unlabeled photos were presented on a large, high-resolution computer monitor, one at a time. For each photo, the scorer assigned a value of 0, 1 or 2 for each of the four RGS action units (see section 3.1 and Figure 1). In every case, a score of "0" indicated high confidence of the scorer that the action unit was absent. A score of "1" indicated either high confidence of a moderate appearance of the action unit, or equivocation over its presence or absence. A score of "2" indicated the detection of an obvious appearance of the action unit, with high confidence.

### Accuracy and reliability determination

A detailed handout was prepared and distributed (by S.G.S.) to members of the J.S.M. lab, explaining each feature and providing prototypic photos for each intensity score (0-2) of each action unit. Five postdoctoral, graduate or undergraduate student coders were then given 104 randomized, unlabeled photos (half *no pain*; half CFA *pain*) in order to assess inter-rater reliability and accuracy of the RGS. Reliability was quantified by comparing average action unit scores across coders, using the intraclass correlation coefficient (ICC) [15]. Accuracy was determined by global *pain *vs. *no pain *dichotomous judgments also made by the scorers.

### Statistical analyses

All statistical analyses were performed using Systat v.11 (SPSS Inc.), with a criterion α = 0.05, except for the ICC, which was calculated using SPSS v. 17. Time-course data were analyzed using repeated measure ANOVA; group/dose differences by one- or two-way ANOVA followed where appropriate by Dunnett's case-comparison posthoc test. Half-maximal analgesic doses (AD_50_s) were calculated using the method of Tallarida and Murray [16] as implemented by FlashCalc 40.1^® ^software (M. Ossipov, University of Arizona).

## Results

### The RGS compared to the MGS

Preliminary attempts to use the existing MGS to score pain in rats were broadly successful (data not shown), but with increasing experience we noticed one striking difference between the "pain face" of the two rodent species. In the mouse, the nose and cheek at baseline have a smooth appearance, whereas in the presence of pain distinct bulges are noted in both the nose and the cheek regions [9]. By contrast, at *baseline *the nose and cheek regions of the rat display distinct bulging, and with pain the bridge of the nose flattens and elongates, causing the whisker pads to flatten. The flattening of normal bulging in the nose and cheek appear to always occur together, such that a single action unit, which we call *Nose/Cheek Flattening*, appears to show the highest correlation with the presence of pain in the rat. This major change renders the RGS much more sensitive and accurate in detecting pain in rats than the MGS.

**Figure 2:**
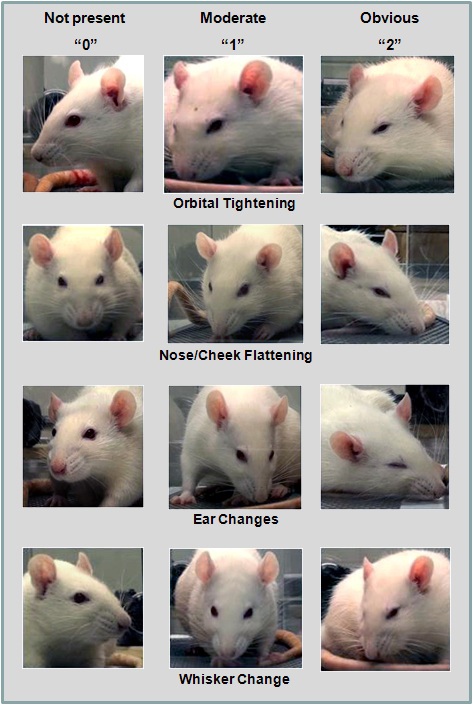
**The four action units of the Rat Grimace Scale (RGS)**. See text for details.

Thus, the *four *action units of the RGS (illustrated in Figure 2) are as follows:

#### 1. *Orbital Tightening*

Rats in pain display a narrowing of the orbital area, manifesting either as (partial or complete) eye closure or eye "squeezing."

#### 2. *Nose/Cheek Flattening*

Rats in pain display successively less bulging of the nose and cheek (see above), with eventual absence of the crease between the cheek and whisker pads.

#### 3. *Ear Changes*

The ears of rats in pain tend to fold, curl and angle forwards or outwards, resulting in a pointed shape. The space between the ears may appear wider.

#### 4. *Whisker Change*

The whiskers of rats in pain move forward (away from the face) from the baseline position, and tend to bunch, giving the appearance of whiskers standing on end.

More detailed descriptions may be found in the RGS training manual, provided as Additional File 1.

### Reliability and accuracy of the RGS

**Figure 3:**
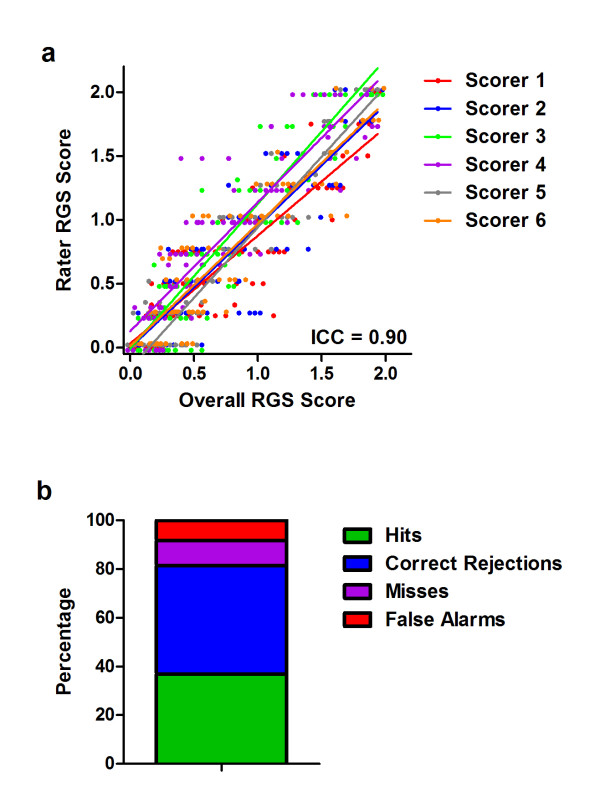
**Interrater reliability (a) and accuracy (b) of the RGS in the quantification of pain**. In both cases 100 photographs were scored, half *pain *(CFA) and half *no pain *(baseline). Scorer 1 developed the RGS, and trained the others; the signal detection data represent the average of all six scorers. Hits: *pain *photograph scored as *pain*; Correct Rejections: *no pain *photograph scored as *no pain*; Misses: *pain *photograph scored as *no pain*; False Alarms: *no pain *photograph scored as *pain*. ICC: intraclass correlation coefficient (see text).

Reliability and accuracy of the RGS in quantifying CFA pain is shown in Figure 3. The overall ICC was 0.90 (Figure 2a), exactly the same as that of the MGS on the abdominal constriction test [9]. Reliability was statistically identical for front-view (two eyes present) versus side-view (one-eye present) photos. All four action units displayed high inter-rater reliability, with ICCs ranging from 0.86 (*Nose/Cheek Flattening*) to 0.96 (*Orbital Tightening*). On average, the scorers achieved an accuracy rate of 81.6% (Figure 2b); of inaccurate *pain*/*no pain *determinations, false alarms (8.2%) were slightly more common than misses (10.3%). Individual scorers' accuracy ranged from 76.0-87.5%. Front-view and side-view photos were scored with equal accuracy.

### Quantification of pain in three nociceptive assays using the RGS

**Figure 4:**
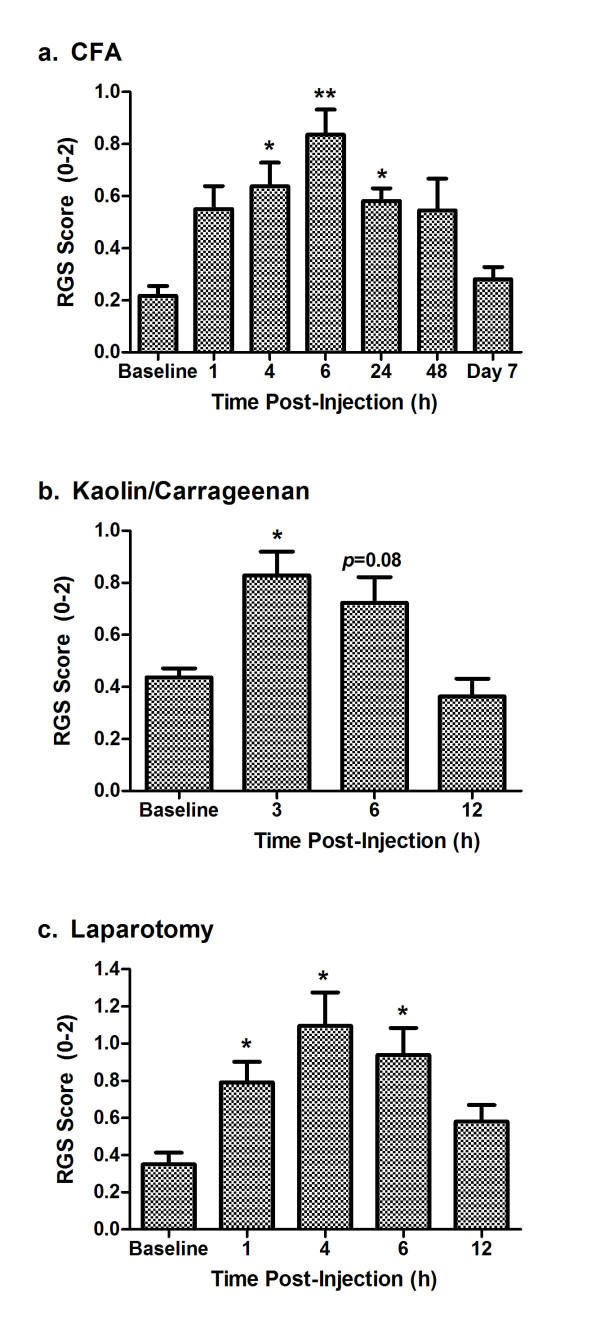
**Quantification of spontaneous pain in three nociceptive assays: intraplantar CFA (a), intraarticular kaolin/carrageenan (b), and postoperative (laparotomy) pain (c)**. Bars represent mean ± SEM RGS score (*n *= 6-10 rats/assay). *p< 0.05; **p< 0.01 compared to baseline (Bonferroni-corrected).

The extent and time course of pain in three nociceptive assays was quantified using the RGS (Figure 4). Baseline RGS scores were the same in each experiment (*F*_2,19 _= 1.7, *p *= 0.21), and in every case RGS scores increased from baseline levels by 2-4-fold, and then returned to baseline levels. Repeated measures ANOVA was performed on RGS scores (including baseline), followed by posthoc testing for repeated measures with Bonferroni correction for multiple comparisons.

In the CFA assay (analyzed up to 24 h), ANOVA revealed a highly significant effect of repeated measures (*F*_4,36 _= 9.2, *p*< 0.001). Significant increases from baseline were observed at 4 h, 6 h and 24 h post-injection. Because the 24-h time point still showed significant grimacing, a separate cohort of rats was tested at 48 h and 7 days post-injection. Although the 48-h time point was significantly increased from its own baseline (*F*_2,14 _= 9.1, *p*< 0.005; posthoc test for repeated measures, *p*< 0.05), increased variability was observed such that grimacing was observed in some rats but not others. In any case, by day 7 all rats had returned to baseline levels.

In the kaolin/carrageenan assay, ANOVA revealed a highly significant effect of repeated measures (*F*_3,15 _= 11.9, *p*< 0.001). A significant increase from baseline was observed at 3 h post-injection, with a strong trend to increased scores (*p *= 0.08) observed at 6 h. RGS scores were at baseline levels by 12 h post-injection.

Finally, in the laparotomy assay, ANOVA revealed a highly significant repeated measures effect (*F*_4,20 _= 8.0, *p*< 0.001). A significant increase from baseline was observed at 1 h, 4 h and 6 h post-injection, but not at 12 h post-injection.

### Individual action units

To compare the utility of the four action units comprising the RGS, we analyzed difference in scores (*pain *- *no pain*) of data from all three assays combined. To facilitate a valid comparison, the single time point showing maximal RGS scores in each assay (6 h for CFA, 3 h for kaolin/carrageenan, and 4 h for laparotomy) was used to supply *pain *photos.

**Figure 5:**
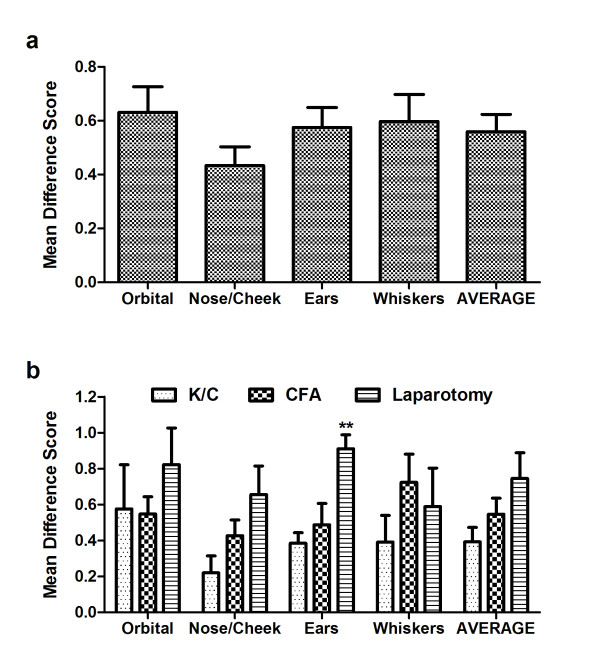
**Prominence of individual action units at the peak of apparent spontaneous pain in each assay (see Figure 4)**. Bars represent mean ± SEM difference scores (*pain *- *no pain*; *n *= 23 rats). Overall, all action units were equally prominent statistically (a), and this was also true in each assay considered separately (b). **p< 0.01 (Bonferroni-corrected) compared to other assays. K/C = kaolin/carrageenan.

No differences in the RGS difference scores were noted among the four action units (one-way ANOVA: *F*_3,63 _= 1.8, *p *= 0.16), attesting to their individual utility (Figure 5a). Correlations between each action unit and the average (i.e., overall) difference scores ranged from *r *= 0.72-0.86 (all *p*≤ 0.001). Averaging all four action units appears to improve the signal-to-noise ratio, as a smaller S.E.M. was observed for the average score (0.06) than for any of the individual action units (0.07-0.10). A comparison of action unit difference scores by nociceptive assay (Figure 5b) revealed only one instance of a significant difference in action unit "strength" between assays: the *Ear Changes *action unit was significantly more prominent after laparotomy than in the other assays (*p*< 0.01; corrected). However, this appears to be just an exaggerated example of a general trend whereby laparotomy produced higher peak RGS scores (see Figure 5b "AVERAGE").

### Morphine Analgesia

**Figure 6:**
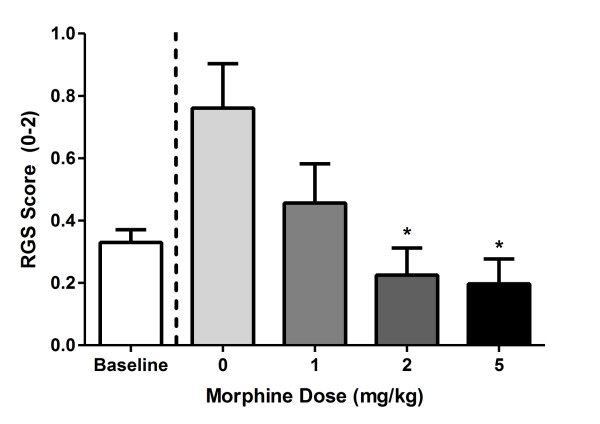
**Quantification of morphine analgesia by the RGS**. Morphine was administered 5.5 h after CFA and 15 min before the start of 30-min digital video recording. Bars represent ± SEM RGS score (*n *= 4-10 rats/dose). *p< 0.05 compared to saline (0) by Dunnett's case-comparison posthoc test (one-way).

If the RGS is truly quantifying pain levels, then it must be able to detect the pain-inhibiting effect of known analgesics such as morphine. Figure 6 shows dose-dependent inhibition (*F*_3,16 _= 4.4, *p*< 0.05) of facial grimacing caused by CFA (at the 6 h time point) by morphine. The AD_50 _of morphine was calculated as 0.8 mg/kg (95% confidence interval: 0.4-1.2 mg/kg).

## Discussion

We report here the development, reliability, accuracy, analgesic sensitivity, and utility of the RGS, a method to quantify spontaneous pain in the laboratory rat. In addition, we have developed an automated system--the RFF software package--that can successfully extract scorable image files from digital video, previously the most labor-intensive step in the application of the RGS (or MGS). The RFF can be obtained directly from one of the authors (ODK at king@bbri.org) upon request by interested individuals, at no charge for academic users and via licensing agreement for corporate users.

### RGS vs. MGS

As predicted by the evolutionary conservation of facial expressions of emotions [8], including pain [7], the "pain face" of the rat was found to be broadly similar to that of the mouse, with three of the RGS action units essentially unchanged from the MGS. A major exception is the nose and cheek, whereby pain in the mouse results in bulging, but in the rat bulging occurs naturally and this characteristic actually diminishes when the rat is in pain.

Observed accuracy rates for the RGS are lower than those observed in the MGS using similar high-definition video (97% by S.G.S.) [9], but still far above chance levels. We note that when testing the accuracy of the MGS, another well-validated pain-related behavior (abdominal constrictions) was used to verify the existence of spontaneous pain in each subject, whereas here, using CFA, we were forced to simply assume its existence. This fact likely accounts for the lower accuracy values obtained, since rats in some of the *pain *photographs may not have been, at that precise moment, actually in pain, which would artificially inflate the miss rate.

Inter-rater reliability of the RGS was very high, as high as on the MGS. We note, however, that this was only tested in five individuals in one laboratory. We encourage others to use the method so that true reliability and accuracy rates can be ascertained.

### Time course of inflammatory pain

Peak RGS scores were observed at 6 h post-CFA, 3 h post-kaolin/carrageenan, and 4 h post-laparotomy. It is tempting to conclude that this represents the peak of spontaneous pain in these assays, as opposed to allodynia. There are, of course, very few extant studies where spontaneously emitted behaviors have been recorded in these assays, and even then it's not clear that what is being measured is spontaneous pain (as opposed to mechanical allodynia), or even pain at all [1,4,5]. In an early study using intraplantar 100% CFA in the rat [17], a number of behavioral characteristics including food intake, open field behavior, and core body temperature were altered by CFA, some for over 5 weeks. In contrast, Djouhri and colleagues [18], using spontaneous foot lifting as a measure of spontaneous pain in Wistar rats, noted that all rats displayed foot lifting 1 day after injection, but less than 20% did by day 2 and none did at 4-7 days post-injection. Using a suite of behaviors (including muscle twitching, back arching, staggering, and abdominal writhing), Roughan and Flecknell [19] concluded that postoperative pain after laparotomy decreased significantly after 3-5 h post-surgery. Using exploratory activity and conditioned operant responding for sucrose pellets as the measure, in contrast, Martin et al. [20] observed changes after surgery lasting up to 2-3 days.

The time course of mechanical allodynia and thermal hyperalgesia in these models is better known, albeit dose- and strain-dependent. The first study to use 50% CFA in the rat observed peak thermal hyperalgesia at 4 h post-injection and a return to baseline by 15 days; mechanical allodynia peaked at 2 days post-injection and was resolved by 5 days [21]. The duration of changes in the other two models is much more limited. Thermal hyperalgesia in the kaolin-carrageenan model was found to peak at 8-12 h and resolve by 2 days post-injection [12]. Electrophysiological experiments have shown that primary afferent fibers in the joint are sensitized in the kaolin-carrageenan model 3-6 h post-injection [22]. After laparotomy in the Wistar rat, mechanical allodynia was noted from 2.5-6.5 h post-surgery [13], although in a recent study (involving in addition to the incision the implantation of a radiotelemetry transmitter) significant allodynia was observed for 9 days [23].

Overall there is good concordance between the time course of inflammatory pain inferred from the literature and our current data. It is important to note, however, that the disappearance of the facial grimacing may not necessarily represent the disappearance of spontaneous pain, as there are adaptive advantages to inhibiting a "pain face" as soon as possible.

### New approaches to algesiometry

The problematic symptoms of chronic pain in humans include spontaneous pain, numbness, dysesthesias, and evoked (mechanical, heat and cold) hypersensitivity. But these are not equally common, or of equal concern. For both neuropathic and non-neuropathic pain, spontaneous or ongoing pain (especially deep pain) is far more prevalent than evoked pain, especially touch- and warmth-evoked pain [24,25]. Spontaneous pain is also rated as more bothersome, and more highly correlated with global ratings of pain severity [24]. Despite this clinical reality, preclinical studies of pain are strongly weighted towards the study of mechanical and thermal hypersensitivity states, largely for reasons of practicality and inertia [4].

However, new approaches to measuring pain (and/or the impact of pain) appear to be gaining popularity; these include thermal preference/escape models [e.g., [26,27]], conditioned place aversion [28,29], conditioned place preference (to pain inhibition) [30,31], and ultrasonic vocalization [32]. Compared to these, facial expression coding has the considerable advantage that no subject training or special equipment (other than a video camera) are required. It also provides the advantage of more complete blinding of the experimenter [33], since during scoring the presence or absence of an inflamed or guarded hind paw is completely obscured. Quantifying pain by facial expression is also the only technique of practical value in veterinary medicine (including laboratory animal welfare), as it can in fact be performed in real time by trained investigators, animal technicians and/or veterinarians.

The major disadvantages to blinded facial expression coding for research purposes are the labor-intensive nature of frame grabbing, a problem now largely solved with RFF software, and the limited duration (< 48 h) of the pain face. This limitation is imposed by the nature of facial grimacing itself, which is also not observed in human chronic pain patients. Thus, the study of real-time spontaneous pain in chronic neuropathic assays awaits the development of a useful dependent measure.

## Competing interests

The authors declare that they have no competing interests.

## Authors' contributions

SGS, RES, AZ, AHT, LJM, JSW, JCSM, SZ, and SZ collected the data. JJM and ODK edited the manuscript. PW and ODK designed the software. JSM conceived of and designed the study (with assistance from JJM), and wrote the draft of the manuscript. All authors read and approved the final manuscript.

## Supplementary Material

Additional file 1 **Rat Grimace Scale (RGS): The Manual**. This training manual describes detailed procedures for the implementation of the RGS. Click here for file
