# Supplementary material for: The Rat Grimace Scale: A partially automated method for quantifying pain in the laboratory rat via facial expressions
Source: Mol Pain. 2011 Jul 29;7:55. doi: 10.1186/1744-8069-7-55 (PMC3163602; doi:10.1186/1744-8069-7-55)
Supplement: Additional file 1 — Rat Grimace Scale (RGS): The Manual. This training manual describes detailed procedures for the implementation of the RGS. [file 1744-8069-7-55-S1.PDF]

RAT GRIMACE SCALE (RGS): THE MANUAL

I. VIDEO & FRAME CAPTURE PROCEDURES:

Place rats individually in cubicles (21 x 10.5 x 9 cm high), with two walls of transparent Plexiglas® and two opaque side walls (to encourage rats to face either forward or backward). Place two digital video cameras immediately outside both Plexiglas walls to maximize opportunity for clear head shots. Higher-resolution color video is highly recommended (e.g., 1920 x 1080, Sony High Definition Handycam® Camcorder)

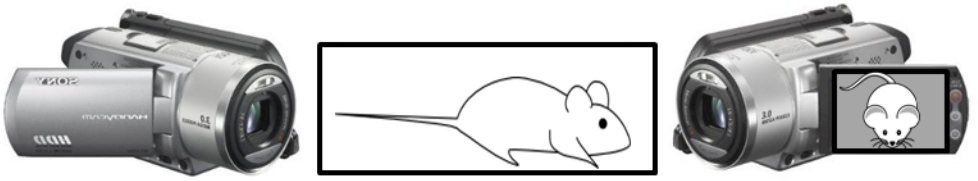

Digitally videotape rats during habituation phase, prior to administration of noxious stimulus (i.e., baseline/"no pain" video). This habituation period should not exceed 30 min, as animals generally fall asleep by this time, and sleeping photos should not be coded. At the appropriate time point after noxious stimulation (i.e., "pain" video), place rats back into cubicle and film for appropriate time; this time will depend on the assay being used.

Using Windows Media Player® or its equivalent, review "no pain" video and capture frames at 3 minute intervals, when a clear, unobstructed head shot is observed. On a 30 minute video, this method would yield 10 baseline photos per animal. (*Note: In Windows Media Player Version 9.0 or less, "Ctrl-I" can be used to grab a frame and save it in JPG [or equivalent, e.g., TIF] format). For "pain" videos, if pain is associated with an overt behavioral response, frames should be grabbed during the exhibition of such behavior, unless it involves purposeful head movement (e.g., licking of the hind paw). For assays which are not associated with any behavioral response, use the same frame capture method as for "no pain" videos, grabbing frames at regular intervals. The grabbed frame should include, primarily the head with most AU visible (i.e., nose, cheek, whiskers, eyes and ears)

The resultant JPG files can be copied into PowerPoint, one photo per slide. A macro can be obtained from the web (www.tushar-mehta.com/powerpoint/randomslideshow/index.htm) in order to randomize slide order.

II. Coding Procedures:

Hide photo identifications (for example by placing them in the Notes panel within PowerPoint) during coding to ensure that coding is performed blind. Coders should always use the computer when coding, as photographs may lose resolution with printing. Before beginning the coding process the coder should view and familiarize oneself with baseline photos provided to determine the specific rat features present. All faces are to be coded for the presence and intensity of the following specific facial Action Units (AU), relative to the status of the region of the baseline prototype.

A. Intensity Ratings:

Intensity ratings are coded for each AU.

AU is not present = 0
AU moderately visible = 1
AU pronounced = 2

An RGS score for each photograph is calculated by averaging intensity ratings for each AU. An RGS difference score (relative to baseline/"no pain") can then be calculated for each subject, and averaged across a group. These calculations are described in detail at the end of the document.

NOTE: If an action unit is not present in the photo, a score of "NA" (Not Applicable) should be used.

B. Action Units:

1. Orbital Tightening

Rats in pain display a narrowing of the orbital area, a tightly closed eyelid, or an eye squeeze. An eye squeeze is defined as the orbital muscles around the eyes being contracted. The nictitating membrane may be visible around the eye and becomes more pronounced as the pain intensifies. As a guideline, any eye closure that reduces the eye size by more than half should be coded as a "2". *Note that sleeping rats display closed eyes, but of a relaxed nature, whereas a rat in pain may display a closed eye with tight orbital muscles. Photographs of sleeping rats should not be taken and/or coded.

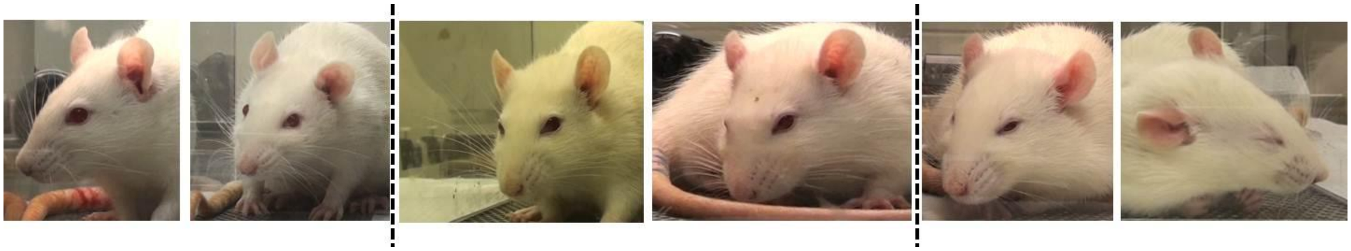

"0" **"1"** **"2"**

2. Nose/Cheek Flattening

Rats in pain display a *lack* of bulge on top of the nose (i.e., a flattening of the nose). In the "no pain" condition a clear bulge is present at the bridge of the nose. The whisker pads are also rounded and slightly puffed out, leaving a clear crease between the pads and the cheek. When in pain, the bridge of the nose flattens and elongates, causing the whisker pads to flatten. At this time the crease between the pads and the cheek is no longer present. In frontal headshots, the nose may appear narrower and longer.

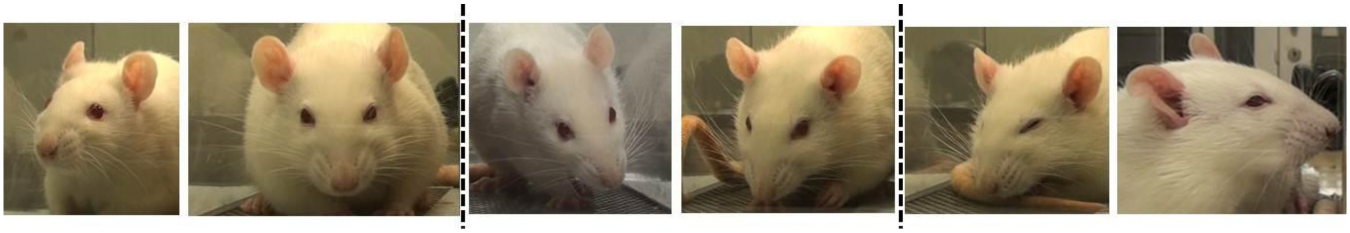

"0" **"1"** **"2"**

3. Ear Changes (Position, Orientation, Shape)

The ears of rats in pain may be curled and pointed more than in the baseline position. In the baseline position ears are roughly perpendicular to the head, face forward, and are angled slightly backward. Importantly, the ears also have a rounded shape. In pain, the ears tend to fold, curl inwards and are angled forward. This curling of the ears tends to result in a "pointed" shape of the ears. In pronounced pain states, the ears are angled outward and are held close to 45° away from both the perpendicular axis and the nose. As a result, the space between the ears may appear wider relative to baseline.

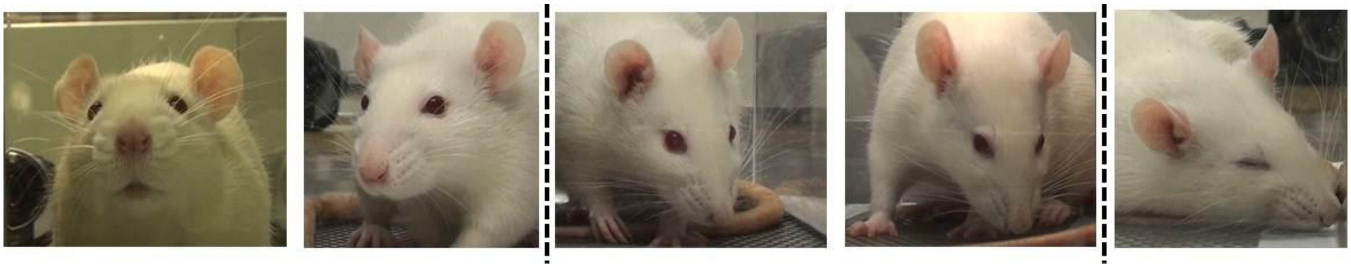

"0" **"1"** **"2"**

4. Whisker Change

Rats in pain have whiskers that have moved from the baseline position and orientation. Whiskers start relaxed and drooping slightly downwards and, as pain progresses, tension in the pads increases and they become angled back along the head. In pain, the whisker pad is contracted causing the whiskers to bunch and be directed outwards away from the face. This gives the appearance of the whiskers as "standing on end". As follicles become tense, whiskers are closer together and are less distinct.

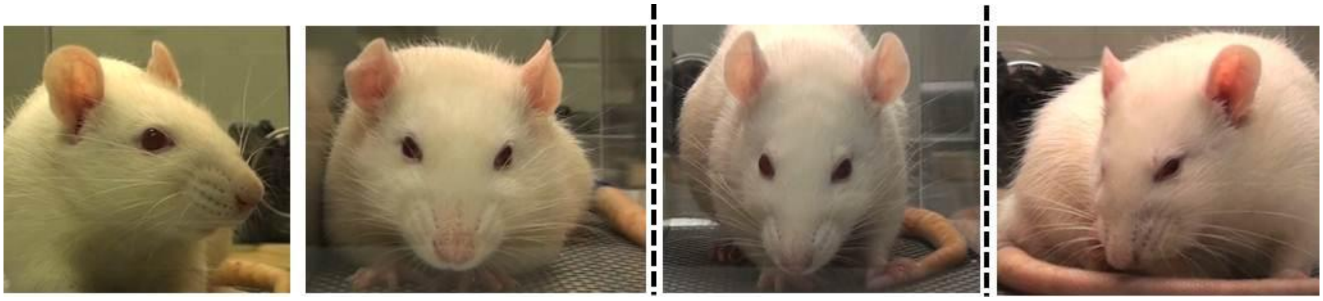

"0" **"1"** **"2"**

III. CALCULATING RGS SCORES:

1. Average all AUs for each photograph to give RGS score. Most of these preliminary calculations are easily carried out using Excel (see example below):

	A	B	C	D	E	F
1	**Photo ID**	**Orbital**	**Nose/Cheek**	**Ears**	**Whiskers**	**RGS**
2	1	2	1	1	2	1.5
3	2	0	0	1	0	0.25
4	3	2	2	2	2	1.75

2. After unblinding, sort data by subject (e.g., "Rat") then by condition. Note that "no pain" photographs are almost always baseline photographs taken prior to noxious stimulation:

3. Calculate mean RGS score for all "no pain" and all "pain" photographs for each subject and subtract the mean for the "no pain" photographs from the mean for the "pain" photographs to give an RGS difference score for each subject:

4. Difference scores can then be averaged across subjects to give a mean difference score, which can be analyzed for significance using a one-sample Student's *t*-test compared to 0.

IV. ATTRIBUTION:

For all uses of the RGS, please attribute our companion publication as follows:
Sotocinal, S.G., Sorge, R.E., Zaloum, A., Tuttle, A.H., Martin, L.J., Wieskopf, J.S., Mapplebeck, J.C.S., Wei, P., Zhan, S., Zhang, S., McDougall, J.J., King, O.D., and Mogil, J.S. The Rat Grimace Scale: a partially automated method for quantifying pain in the laboratory rat via facial expressions. *Mol. Pain*, in press.

Please address all questions to Dr. Jeffrey S. Mogil at jeffrey.mogil@mcgill.ca.

Good luck!
